# Supplementary figures and images for: Bone mesenchymal stem cell-derived exosomes prevent hyperoxia-induced apoptosis of primary type II alveolar epithelial cells in vitro
Source: PeerJ. 2022 Sep 2;10:e13692. doi: 10.7717/peerj.13692 (PMC9443791; doi:10.7717/peerj.13692)

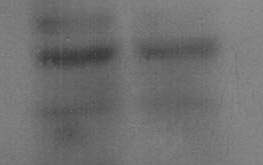

Supplement: Supplemental Information 3 [file peerj-10-13692-s003.zip › original figures/CD63.jpg]

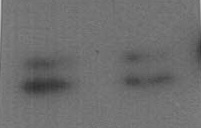

Supplement: Supplemental Information 3 [file peerj-10-13692-s003.zip › original figures/CD9.jpg]

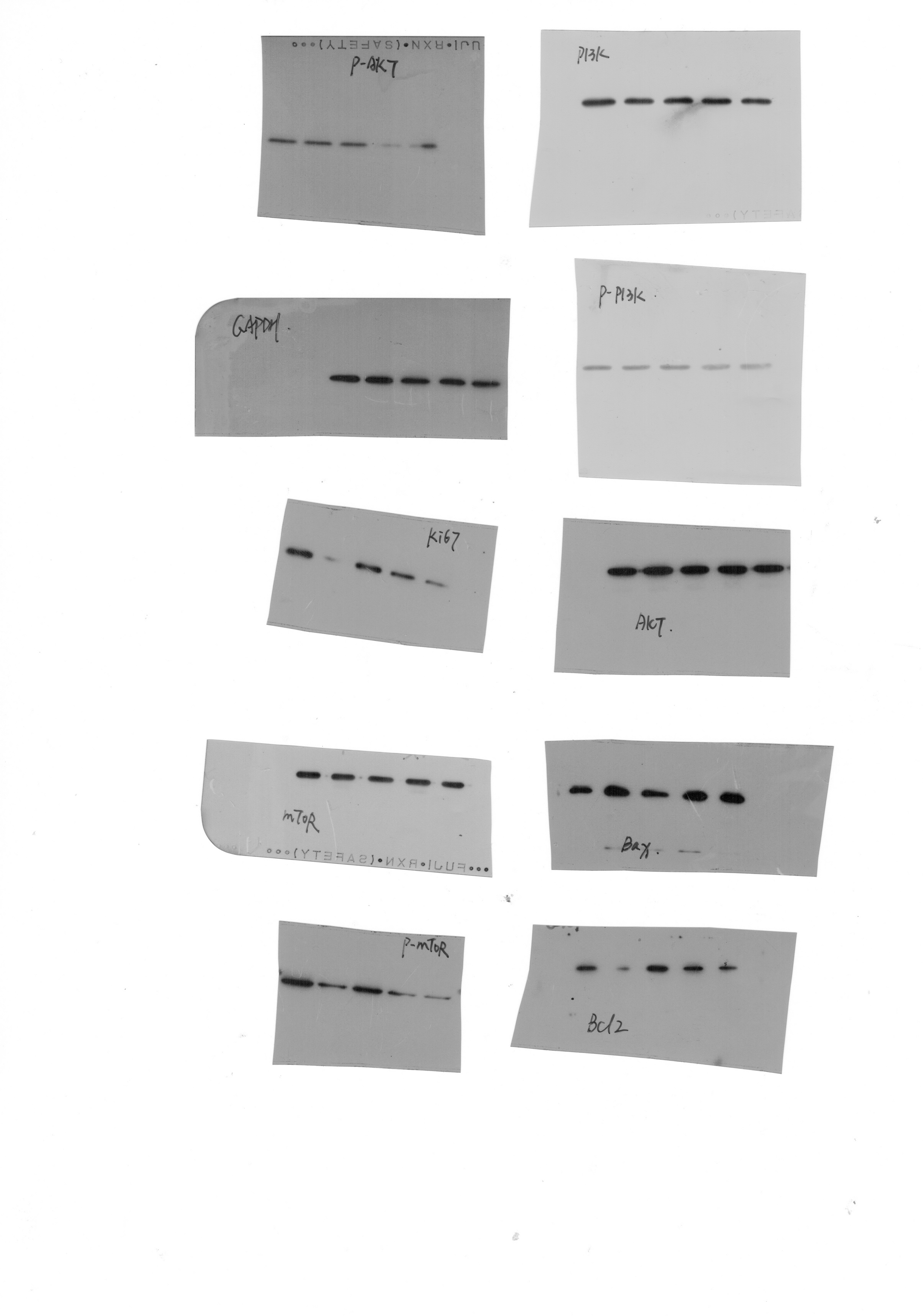

Supplement: Supplemental Information 3 [file peerj-10-13692-s003.zip › original figures/Original Whole Gel(s) for Western Blots1.jpg]

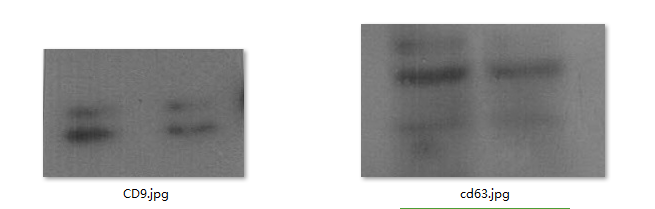

Supplement: Supplemental Information 3 [file peerj-10-13692-s003.zip › original figures/Original Whole Gel(s) for Western Blots2.png]
